# Factors contributing to variability in metformin concentration in polycystic ovary syndrome

**DOI:** 10.3325/cmj.2024.65.138

**Published:** 2024-04

**Authors:** Valentina N. Nikolić, Milan Stefanović, Dejan Mitić, Slavica Sunarić, Maša Jović, Hristina Trajković, Sanja Stanković, Aleksandra Ignjatović, Dragana Stokanović

**Affiliations:** 1 Department of Pharmacology with Toxicology, University of Niš Faculty of Medicine, Niš, Serbia; 2 Department of Gynaecology and Obstetrics, University of Niš Faculty of Medicine, Niš, Serbia; 3 Gynaecology and Obstetrics Clinic, University Clinical Centre Niš, Niš, Serbia; 4 Department of Chemistry, University of Niš Faculty of Medicine, Niš, Serbia; 5 Department of Pharmacy, University of Niš Faculty of Medicine, Niš, Serbia; 6 Department of Medical Statistics and Informatics, University of Niš Faculty of Medicine, Niš, Serbia; * These authors contributed equally.

## Abstract

**Aim:**

To investigate the factors affecting metformin concentrations after chronic administration in patients with polycystic ovary syndrome (PCOS), focusing on the pharmacokinetic variability and its implications for personalized therapy.

**Methods:**

This study enrolled 53 PCOS patients undergoing long-term metformin treatment at the Clinic for Gynecology and Obstetrics in Niš, Serbia, from February to December 2019. Pharmacokinetic parameters were measured from blood samples, and metformin concentrations were determined with validated analytical techniques.

**Results:**

There was a significant variability in metformin concentrations among PCOS patients, with body mass index (BMI) identified as a major influencing factor. Higher BMI was associated with lower plasma metformin levels, a finding suggesting an altered pharmacokinetic profile in obese patients.

**Conclusions:**

This study highlights the critical role of BMI in influencing metformin pharmacokinetics in PCOS patients and underscores the need for personalized treatment strategies in patients with PCOS.

Polycystic ovary syndrome (PCOS) is a complex endocrine disorder affecting 5%-18% of women worldwide, characterized by a spectrum of reproductive, metabolic, and psychological effects (1). Its diagnostic requirements, based on the 2003 Rotterdam criteria, include hyperandrogenism, irregular menstrual cycles, and polycystic ovarian morphology, which underscores the condition's heterogeneity (2). Beyond its primary symptoms, PCOS is intrinsically linked to metabolic disturbances such as insulin resistance and dyslipidemia, increasing the risk of type 2 diabetes and cardiovascular disease. Moreover, the metabolic alterations characterized by insulin resistance and hyperandrogenemia, coupled with chronic low-grade inflammation, are directly linked to an elevated risk of cardiovascular disease (3).

Metformin is recommended as the first-line treatment for type 2 diabetes according to guidelines from both American and European diabetes associations, owing to its proven safety, effectiveness in reducing HbA1c levels, and a minimal risk of hypoglycemia (4). Its application has extended to reproductive health, particularly for PCOS management, due to its impact on insulin resistance and hormonal imbalances. While metformin effectively reduces testosterone levels in PCOS (5), its effects on menstrual irregularity and hyperandrogenism symptoms are variable. It moderately improves ovulation rates but is less effective than clomiphene or letrozole in increasing pregnancy and birth rates (6), which makes it a secondary treatment option for anovulation (7). Recent studies have suggested a potential improvement in pregnancy rates among non-obese women with PCOS (8), but further research is needed to fully understand metformin's efficacy in this area. Although metformin can lower BMI and certain hormonal levels in overweight PCOS patients (9,10), its impact on other metabolic markers remains inconclusive.

Metformin has an oral bioavailability of around 55%, with elimination occurring primarily through the kidneys via glomerular filtration and tubular secretion, in an unchanged state. The absorption, hepatic uptake, and renal excretion of metformin are notably enhanced by the action of organic cation transporters (11).

Despite its widespread use, the variability in patient response and the precise determinants of metformin's pharmacokinetics in PCOS remain inadequately understood and factors influencing metformin levels in PCOS have not been identified. Therefore, this study aimed to investigate the factors influencing metformin levels in PCOS patients. Addressing this issue is crucial, especially considering the risk of metformin-associated lactic acidosis, even in patients without chronic renal impairment (12).

## METHODS

### Patients and methods

The study was conducted at the Clinic for Gynecology and Obstetrics, University Clinical Center of Niš, Serbia, from February to December 2019. It was approved by the Ethics Committee of the University Clinical Center in Niš (No. 38212). All patients were briefed on the study protocol and provided informed written consent. The inclusion criteria were age over 18 years, PCOS diagnosis made by a gynecologist according to established diagnostic criteria, and ongoing metformin treatment. The non-inclusion criteria encompassed pregnancy, lactation, mental health disorders, and refusal to participate.

We enrolled 53 patients with PCOS, all of whom were preparing for *in vitro *fertilization (IVF). Additionally, a subset of these patients (20.75%) received bromocriptine for the treatment of hyperprolactinemia. This setup reflects the complex clinical scenarios encountered in PCOS management and enhances the study’s generalizability to real-world settings. The median age of participants in our study was 33 years (range 23-43 years). Data on clinical and demographic variables, comorbidities, total body weight, and age were extracted from medical records. Lifestyle habits were assessed through patient interviews. Two blood samples were collected from each participant: one for routine laboratory analyses and one to measure the drug's concentration. This sample was obtained just before patients took the next dose of metformin, more than a month after initiating therapy, because we assumed that steady state concentrations were achieved. Metformin levels were determined with high-performance liquid chromatography (HPLC), which ensured precise quantification.

### Determination of metformin concentration

HPLC analysis of metformin was conducted with an Agilent Technologies 1200 Series apparatus with a DAD detector (Agilent, Santa Clara, CA, USA). The analysis was performed with the chromatographic column Zorbax NH2 (4.6 × 150 mm, 5.0 μm) by Agilent Technologies. Agilent ChemStation program was used for monitoring the chromatographic system and data acquisition.

Metformin hydrochloride, certified reference material by Sigma-Aldrich (St. Louis, MO, USA) was used. Acetonitrile and methanol were of HPLC and LC-MS grade (Honeywell Riedel-de Haen, Offenbach, Germany).

HPLC analysis of metformin in blood samples was performed using a slightly modified method published by Mary Rebecca et al (13). The analysis was carried out at 30 °C on Zorbax-NH2 column. The mobile phase consisted of 100% acetonitrile with a flow rate of 0.725 mL/min. Detection was performed at 232 nm, and quantification was done with calibration curve method. A sample of 200 μL of blood plasma was mixed with 200 μL of acetonitrile. The mixture was vortexed and centrifuged for 10 min at 4 °C and 14 000 rpm. The supernatant was filtered through 0.45 μm syringe filter, and 20 μL was injected on Zorbax-NH2 column.

### Statistical analysis

Continuous data are presented as mean with standard deviation, and categorical variables as counts and percentages. The Shapiro–Wilk test was used for normality testing. The differences between the groups were evaluated with a *t* test or a Mann-Whitney U test. Multivariate regression analysis was performed to examine factors influencing metformin concentration. The statistical significance level was set at *P* < 0.05. The analysis was conducted with SPSS v. 27.0 (IBM Corp., Armonk, NY, USA).

## RESULTS

**Figure 1:**
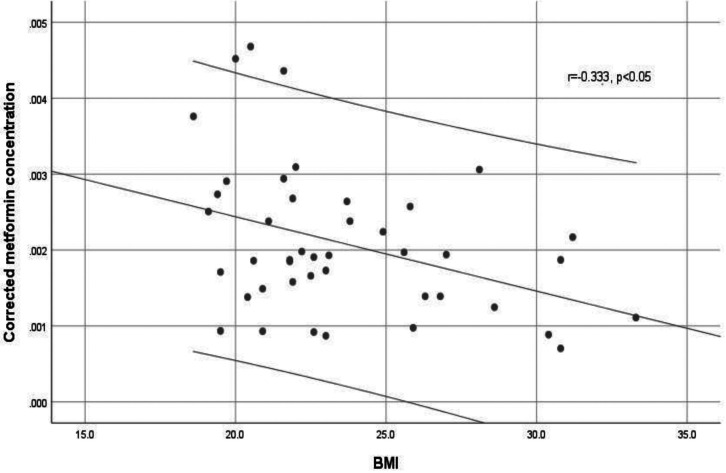
Correlation between corrected metformin concentration and body mass index (BMI).

Descriptive statistics of the examined parameters and laboratory findings in women with PCOS are shown in Table 1. Since the daily dose of metformin varied from 500 mg to 2500 mg, metformin concentration was adjusted for analytical purposes. The influence of age, smoking, BMI, and creatinine clearance on the adjusted concentration of metformin was assessed with standard multiple regression analysis. The tested model explained 33.8% of the variance of the drug concentration. BMI was singled out as an independent predictor (Beta = -0.457, *P* = 0.032) (Table 2, Figure 1). As BMI increased, the corrected drug concentration decreased. This parameter explained 13.6% of the variance of the corrected drug concentration.

**Table 1:** The examined parameters and laboratory findings

Parameter	Mean ± standard deviation (Min-Max) or N (%)
Age (age)	32.08 ± 4.41 (23-43)
Body mass index	23.03 ± 2.96 (19.1-30.8)
Number of patients who received bromocriptine (%)	11 (20.8)
Number of patients with hypothyroidism	12 (22.6)
Number of patients with hyperthyroidism	3 (5.7)
Duration of therapy (months)	12.19 ± 12.59 (1-72)
Metformin daily dose (mg)	1166.67 ± 577.35 (500-2500)
Glucose (mmol/L)	4.93 ± 0.69 (3.9-7.3)
Urea (mmol/L)	3.80 ± 1.13 (1.5-6.4)
Creatinine clearance (mL/min)	111.50 ± 28.35 (71.2-186.2)
Albumin (g/dL)	43.50 ± 0.71 (43.0-44.0)
Cholesterol (total) (mmol/L)	5.25 ± 0.95 (3.49-7.97)
High-density lipoprotein cholesterol (mmol/L)	1.47 ± 0.36 (0.63-2.52)
Low-density lipoprotein cholesterol (mmol/L)	3.18 ± 0.90 (1.31-5.50)
Triglycerides (mmol/L)	1.34 ± 0.74 (0.52-4.16)
Aspartate aminotransferase, (U/L)	17.15 ± 4.37 (11-28)
Alanine amiotransferase (U/L)	18.72 ± 13.08 (7-81)
C-reactive protein(mg/L)	4.20 ± 3.88 (0.3-20.5)
Glycated hemoglobin	31.41 ± 2.82 (26.8-38.0)
Leukocytes (10^9^/L)	7.54 ± 2.12 (4.5-12.7)
Lymphocytes (%)	31.82 ± 6.97 (16.8-47.2)
Monocytes (%)	5.37 ± 1.87 (1.9-10.6)
Granulocytes (%)	65.53 ± 8.15 (53.5-80.4)
Neutrophils (10^9^/L)	4.24 ± 1.57 (2.27-7.99)
Erythrocytes (10^12^/L)	4.55 ± 0.9 (3.91-6.08)
Hemoglobin (g/L)	132.28 ± 10.20 (110.0-164.0)
Hematocrit	0.40 ± 0.03 (0.33-0.47)
Mean corpuscular volume (fL)	88.63 ± 5.16 (70.5-96.5)
Mean corpuscular hemoglobin (pg)	29.03 ± 2.52 (20.1-32.8)
Mean corpuscular hemoglobin concentration (g/L)	328.92 ± 12.55 (298-351)
Red cell distribution width (%)	12.13 ± 0.94 (10.4-15.4)
Platelets (10^9^/L)	261.06 ± 1.52 (162-485)
Mean platelet volume (fL)	8.34 ± 1.14 (6.4-12.3)
Platelet distribution width (%)	18.9 ± 2.12 (15.4-21.8)
Metformin (μmol/L)	14.6 ± 2.2 (9.6-18.4)
Smoking	20 (37.7%)

**Table 2:** Factors influencing the corrected concentration of metformin*

	Unstandardized coefficients		Standardized coefficients	*P* value
	B	standard error	Beta	
Constant	0.060	0.009		<0.001
Age	<0.001	<0.001	-0.182	0.274
Smoking	-0.001	0.002	-0.109	0.526
Body mass index	-0.001	<0.001	-0.457	0.032
Creatinine clearance	-0.001	0.000	-0.061	0.790

*R^2^ adjusted 0.338.

Patients with BMI≥25 had significantly higher systolic blood pressure (*P* = 0.035), glucose (*P* = 0.049), and triglyceride levels (*P* = 0.004). Besides, their daily dose (*P* = 0.034) and the corrected concentration of metformin were significantly higher (*P* < 0.001). There was a significant difference in creatinine clearance between the groups (*P* < 0.001) (Table 3).

**Table 3:** Examined parameters in patients with body mass index (BMI)<25 and BMI≥25

	BMI<25	BMI≥25	p
Age (years)	32.06 ± 3.42	32.86 ± 5.89	0.572
Systolic pressure (mmHg)	110.42 ± 8.65	120.00 ± 9.26	0.035
Diastolic pressure (mmHg)	80.83 ± 17.82	75.63 ± 9.80	0.463
Metformin dose (mg)	991.67 ± 442.77	1326.92 ± 607.12	0.049
Glucose (mmol/L)	4.8 ± 0.66	5.29 ± 0.75	0.034
Urea (mmol/L)	3.72 ± 1.12	3.84 ± 0.91	0.449
Creatinine clearance (mL/min)	102.04 ± 42.67	133.81 ± 29.75	<0.001
Cholesterol	5.16 ± 0.89	5.48 ± 1.09	0.289
High-density lipoprotein cholesterol (mmol/L)	1.55 ± 0.3	1.39 ± 0.46	0.165
Low-density lipoprotein cholesterol (mmol/L)	3.13 ± 0.91	3.30 ± 0.91	0.564
Triglycerides (mmol/L)	1.05 ± 0.3	1.87 ± 1.07	0.004
Aspartate aminotransferase (U/L)	16.64 ± 3.71	18.07 ± 5.72	0.399
Alanine aminotransferase (U/L)	15.67 ± 8.33	24.79 ± 19.3	0.109
C-reactive protein (mg/L)	3.74 ± 4.22	5.06 ± 3.01	0.064
Metformin concentration, corrected	0.248 ± 0.039	0.186 ± 0.031	<0.001

## DISCUSSION

In our study, age, smoking status, BMI, and creatinine clearance collectively accounted for 33.8% of the variability in metformin levels, with BMI emerging as a significant independent predictor.

Since our analysis used daily dose-corrected metformin concentrations, actual concentrations might be lower in heavier patients receiving higher doses. This discrepancy is due to body weight influencing the drug's volume of distribution and clearance rate. Although increasing the dose in patients with higher body weight is intended to maintain therapeutic metformin levels, absolute plasma concentrations may still be lower. This is because the drug's volume of distribution expands with body weight, potentially necessitating higher doses for the same efficacy. Adjusting metformin concentration based on daily dose facilitates efficacy comparison across individuals with varying doses and body weights. However, it is vital to recognize the complexity of the relationship between dose, body weight, and plasma concentration. This relationship is also affected by insulin resistance and renal function.

This study showed a negative correlation between BMI increments and the adjusted metformin concentration. Despite the absence of a confirmed link between metformin concentration and its therapeutic efficacy in previous research (14), the identified relationship between BMI and drug concentration gains specific relevance. This is particularly important given the established and strong association between BMI and the parameters of blood pressure as well as clinical manifestations observed in women with PCOS (15).

The lack of correlation between metformin concentration and its therapeutic efficacy (14) might be partially attributable to the presence of a distinct and profound depot of metformin within erythrocytes. This reservoir could modulate the bioavailability of metformin, thereby influencing its pharmacodynamic effects independent of plasma concentrations. Initial research highlighted the disparity in metformin concentrations between plasma and erythrocytes following acute administration, underscoring a distinct elimination profile in erythrocytes, which is crucial for understanding the drug's overall pharmacokinetics (16). Further investigations in patients with type 2 diabetes mellitus demonstrated metformin's tendency to accumulate in erythrocytes, suggesting a deeper compartmentalization that necessitates dose adjustments based on renal function (17). Expanding beyond diabetes, another study in lung cancer patients showed significant individual variation in metformin concentrations across tissues, emphasizing the importance of exploring metformin's distribution in different indications (18). Additionally, research in prediabetic individuals found that a lower dose could achieve therapeutic serum concentrations, with a noted correlation between serum levels and body weight, opening new avenues for metformin's use and dosage considerations in non-diabetic conditions (19). These findings underscore the complexity of metformin's pharmacokinetics and the need for tailored therapeutic strategies across different patient populations.

Only de Oliveira Baraldi et al investigated the pharmacokinetics of metformin in PCOS patients (20). They determined specific pharmacokinetic parameters of metformin, such as its half-life, time to maximum concentration, and volume of distribution, in a population of nondiabetic pregnant women with PCOS. The study confirmed that metformin oral clearance was increased in nondiabetic pregnant women with PCOS compared with nonpregnant healthy volunteers or diabetic patients, which resulted in lower plasma metformin concentrations. These results are presumably determined by physiological changes during pregnancy, such as increased renal plasma flow (21) and possibly enhanced renal expression or function of organic cation transporter 2 (22), as factors contributing to the increased rate of metformin clearance. As a lower dose is effective for the treatment of prediabetes (19), the question arises whether it is necessary to adjust the dose in pregnant women with PCOS, bearing in mind that they have a faster elimination of the drug. This assumption highlights the crucial need to identify optimal markers of individual drug responses instead of the traditional one-size-fits-all dosing approach toward a more tailored and efficacious treatment paradigm.

The identification of BMI as a critical factor in metformin pharmacokinetics aligns with contemporary research, underscoring the impact of body composition and renal function on the drug's pharmacological profile (23-25). The evidence synthesis highlights the critical role of BMI and other factors such as lean body weight (LBW) and creatinine clearance in optimizing metformin dosing. LBW is a more accurate predictor of metformin pharmacokinetics than total or ideal body weight (23). This emphasizes the need to consider body composition, especially active components, in the pharmacokinetics of metformin in the obese population with type 2 diabetes mellitus, to accurately assess drug distribution and clearance.

The increase in metformin clearance observed in obese patients (26) can be attributed to obesity-related physiological changes that enhance renal function, specifically through increased glomerular filtration rate and possibly tubular secretion (27). This mechanism, driven by obesity-induced hyperfiltration and elevated renal plasma flow, underscores the need for careful consideration of dosing strategies in obese patients. Metformin's renal clearance can be accurately predicted by creatinine clearance, calculated using LBW (28), which underlines the importance of considering both factors when optimizing metformin dosing for individual patients, particularly for ensuring therapeutic efficacy and minimizing the risk of adverse effects.

Hess et al (29) explained the complexity of metformin pharmacokinetics by a lack of direct correlation between metformin blood levels and dose or glomerular filtration rate, except for higher doses (daily dose greater than 1700 mg), for which a significant strong correlation was confirmed (29). In contrast, Duong et al (28) emphasized the influence of renal function on metformin kinetics across all dosing levels, proposing a dosing algorithm based on renal function to maintain consistent drug exposure. In addition to the factors we explored, Duong et al (28) provided an essential perspective on metformin dosing algorithms that is particularly relevant to our findings. Their study proposes a dosing algorithm based on renal function to maintain consistent drug exposure - a critical consideration that complements our observation of BMI as a significant factor influencing metformin pharmacokinetics in PCOS patients. This interplay between renal function and body mass underscores the need for a multifaceted approach to metformin dosing. Specifically, while our results highlight the role of BMI, incorporating renal function assessments as suggested by Duong et al could further refine dosing strategies and ensure effective metformin therapy tailored to individual patient profiles.

Metformin pharmacokinetics is also altered by transporter genetic variability, which affects how the drug is absorbed, distributed, and excreted. Metformin's cellular uptake and elimination are influenced by variants in the genes encoding for transporters, such as organic cation transporter 1 (*OCT1*) and multidrug and toxin extruders (*MATE*). For example, certain *OCT1* variants can reduce metformin's entry into liver cells, potentially decreasing its efficacy. Similarly, variations in *MATE* genes can affect how efficiently metformin is excreted through the kidneys. These genetic differences can lead to considerable interindividual variations in drug response (30).

Türk et al showed that daily variations in the pharmacokinetics of metformin among diabetic patients were influenced by circadian rhythms in glomerular filtration rate, renal plasma flow, and the activity of the organic cation transporter 2 (31). This finding emphasizes the importance of considering biological rhythms in optimizing metformin dosing. Although not explicitly analyzed in this study, the impact of physical activity and dietary habits on insulin resistance and body fat composition could modify the pharmacokinetics of metformin, a topic that warrants further investigation to refine PCOS treatment protocols.

A limitation of our study is the sample size, which may not sufficiently capture the broad variability in PCOS manifestations and may limit the generalizability of the findings to the wider PCOS population. This restriction is particularly pertinent given that the study was conducted in a single center with patients preparing for IVF, which is a group of patients that may not reflect the broader PCOS community. Future studies involving a larger, more diverse sample are recommended to enhance the statistical power and applicability of the conclusions. Another limitation is the absence of non-PCOS, diabetic positive controls, which would have enabled a clearer analysis of metformin's effects independent of PCOS-related hormonal imbalances. We did not evaluate the role of genetic variations in key metformin transporters and related genes, which underscores the necessity for further research to thoroughly understand metformin's pharmacokinetics in individuals with PCOS and potentially lead to customized treatment approaches. Future studies should include such controls and explore the impact of transporter gene polymorphisms, alongside conducting dose-response studies to accurately establish metformin's therapeutic concentration range for PCOS management. This approach aims to refine treatment protocols and enhance patient outcomes, optimizing treatment strategies based on our findings and previous studies.
